# Hydrazine-Induced Sulfur Vacancies Promote Interfacial Charge Redistribution in ZnS/Gel-Derived TiO_2_ for Enhanced CO_2_ Activation and Methanation

**DOI:** 10.3390/gels12010039

**Published:** 2025-12-31

**Authors:** Zhongwei Zhang, Shuai Liu, Jiefeng Yan, Yang Meng, Dongming Hu, Fuyan Gao

**Affiliations:** 1 School of Mechanical Engineering, Zhejiang Sci-Tech University, Hangzhou 310018, China; 2 School of Mechatronics and Energy Engineering, NingboTech University, Ningbo 315100, China; 3 College of Science & Technology, Ningbo University, Ningbo 315100, China; 4 Ningbo Key Laboratory of Urban Environmental Pollution Control, CAS Haixi Industrial Technology Innvation Center in Beilun, Ningbo 315830, China

**Keywords:** photocatalytic CO_2_ reduction, ZnS/TiO_2_, sulfur vacancies, hydrazine hydrate

## Abstract

Defect engineering in semiconductor heterojunctions offers a promising route for enhancing the selectivity of photocatalytic CO_2_ conversion. In this work, a ZnS/gel-derived TiO_2_ photocatalyst featuring sulfur vacancies introduced via hydrazine hydrate (N_2_H_4_) treatment is developed. XRD, HRTEM, and XPS analyses confirm the formation of a crystalline heterointerface and a defect-rich ZnS surface, enabling effective interfacial electronic modulation. The optimized ZnS/gel-derived TiO_2_-0.48 composite achieves CH_4_ and CO yields of 6.76 and 14.47 μmol·g^−1^·h^−1^, respectively, with a CH_4_ selectivity of 31.8% and an electron selectivity of 65.1%, clearly outperforming pristine TiO_2_ and the corresponding single-component catalysts under identical conditions. Photoluminescence quenching, enhanced photocurrent response, and reduced charge-transfer resistance indicate significantly improved interfacial charge separation. Mott–Schottky analysis combined with optical bandgap measurements reveals pronounced interfacial charge redistribution in the composite system. Considering the intrinsic band structure of ZnS and gel-derived TiO_2_, a Z-scheme-compatible interfacial charge migration model is proposed, in which photogenerated electrons with strong reductive power are preferentially retained on ZnS, while holes with strong oxidative capability remain on gel-derived TiO_2_. This charge migration pathway preserves high redox potentials, facilitating multi-electron CO_2_ methanation and water oxidation. Density functional theory calculations further demonstrate that sulfur vacancies stabilize *COOH and *CO intermediates and reduce the energy barrier for *COOH formation from +0.51 eV to +0.21 eV, thereby promoting CO_2_ activation and CH_4_ formation. These results reveal that sulfur vacancies not only activate CO_2_ molecules but also regulate interfacial charge migration behavior. This work provides a synergistic strategy combining defect engineering and interfacial electronic modulation to enhance selectivity and mechanistic understanding in CO_2_-to-CH_4_ photoconversion.

## 1. Introduction

The increasing severity of global climate change has made carbon dioxide (CO_2_) emissions one of the most critical environmental challenges worldwide. As one of the primary greenhouse gases, CO_2_ contributes significantly to global warming, climate anomalies, and ecosystem instability. To address this issue, the development of efficient CO_2_ reduction technologies has become imperative. Photocatalytic CO_2_ reduction has emerged as a promising solution with significant potential. This process utilizes solar energy to convert CO_2_ into valuable chemical fuels such as methane (CH_4_) [1,2], ethane (C_2_H_4_) [3,4], methanol (CH_3_OH) [5,6], and carbon monoxide (CO) [7,8], which not only helps mitigate the environmental burden of CO_2_ but also provides new pathways for clean energy production [9,10].

However, several technical challenges must be overcome for the practical implementation of photocatalytic CO_2_ reduction [11,12]. First, traditional photocatalysts such as titanium dioxide (TiO_2_) have limited application in CO_2_ reduction due to their wide bandgap (~3.4 eV), which restricts their absorption of solar light to the ultraviolet region, comprising only about 5% of the solar spectrum, thereby limiting photocatalytic efficiency [13]. Furthermore, TiO_2_ suffers from significant electron–hole recombination during photocatalytic processes, which further reduces its catalytic efficiency [14].

To overcome these limitations, researchers have proposed a strategy to enhance TiO_2_ photocatalytic performance by constructing semiconductor heterojunctions [15,16]. Materials with narrower bandgaps, such as zinc sulfide (ZnS), have been coupled with TiO_2_ to extend the light absorption range into the visible spectrum and enhance photocatalytic efficiency [17,18]. ZnS, with a bandgap of approximately 3.7 eV, can significantly expand TiO_2_ light absorption range and improve the efficiency of solar energy utilization [17]. The combination of ZnS and TiO_2_ would not only improve the separation of photogenerated electron–hole pairs but also suppress electron–hole recombination, facilitating electron transfer and migration, thereby enhancing CO_2_ activation and reduction. Moreover, surface defects in ZnS, such as sulfur vacancies, have been shown to significantly improve catalytic performance by increasing the separation efficiency of charge carriers and thereby enhancing the selectivity for CO_2_ reduction, particularly for methane (CH_4_) production [19,20]. However, the selectivity of CH_4_ remains very low.

For the sulfur vacancies, the types, concentration, distribution, and formation mechanisms of these sulfur vacancies can vary depending on the material and synthesis method, and these factors can have a significant impact on the final catalytic performance. This variability remains one of the main challenges in this field. Moreover, the introduction of sulfur vacancies is highly dependent on the method of synthesis. Various techniques, including chemical vapor deposition [21], solvothermal methods [22], and atmospheric control, have been employed to generate sulfur vacancies, with each method influencing the concentration and distribution of vacancies in different ways. The concentration and distribution density of sulfur vacancies directly affect the electronic structure and surface activity of the material, thereby influencing its catalytic performance [23]. Therefore, optimizing the synthesis process to precisely control the concentration and distribution of sulfur vacancies is a critical issue for improving the performance of ZnS/TiO_2_ composite photocatalysts.

Additionally, the interfacial structure of ZnS/TiO_2_ heterojunctions plays a crucial role in the catalytic performance. The heterojunction between TiO_2_ and ZnS facilitates the migration of photogenerated electrons from ZnS to the conduction band of TiO_2_, reducing electron–hole recombination and enhancing photocatalytic activity [17,18,24,25,26]. However, the interfacial properties of TiO_2_ and ZnS remain a complex topic. The formation of the heterojunction, the distribution of interfacial electrons, and the types of interfacial defects all affect the migration efficiency of charge carriers. Therefore, further investigation into the interfacial structure of ZnS/TiO_2_ heterojunctions and optimization of their interface characteristics are essential for improving their photocatalytic performance.

Another key factor influencing photocatalytic performance is the method of catalyst synthesis. Defects, surface structure, and elemental doping in the catalyst all significantly affect its catalytic performance [17,21]. Existing research indicates that controlling the surface and structural defects of materials through optimized synthesis methods can enhance photocatalytic activity. Specifically, defect engineering in semiconducting materials such as oxides and sulfides is crucial for improving photocatalytic efficiency [21,27]. Introducing controlled amounts of defects, such as oxygen vacancies or sulfur vacancies, can significantly improve the separation and transport of charge carriers, thereby enhancing the efficiency of photocatalytic reactions.

In recent years, hydrazine hydrate (N_2_H_4_) has gained widespread use as a green reducing agent in the defect engineering of materials [28] and catalyst synthesis [29]. Hydrazine hydrate has strong reducing properties and can effectively introduce defects on the surface of semiconductors, especially oxygen and sulfur vacancies in oxides [28,30] and sulfides [31], under mild conditions. Being a low-toxicity, environmentally friendly reducing agent, hydrazine hydrate avoids the environmental pollution associated with traditional toxic reducing agents, thus aligning with the principles of green chemistry. Hydrazine hydrate has been shown to generate sulfur vacancies and quantum dots in ZnS [32,33], the use of hydrazine hydrate as a reducing agent not only improves the catalytic performance of the material but also enhances its stability, allowing the catalyst to maintain high activity over multiple cycles.

Thus, this study aims to investigate the application of hydrazine-modified ZnS/gel-derived TiO_2_ composite materials in photocatalytic CO_2_ reduction. The focus of the research includes exploring the effects of hydrazine modification on the surface defects of ZnS, and how optimizing the ratio of ZnS to gel-derived TiO_2_ can further improve photocatalytic performance and product selectivity. Through a combination of experimental and theoretical approaches, this study will contribute to the understanding of the potential of hydrazine as a green reducing agent in material synthesis, and provide new insights for the design of catalysts for photocatalytic CO_2_ reduction.

## 2. Results and Discussion

### 2.1. Characterization of Phase, Morphology, and Element Distribution

**Figure 1:**
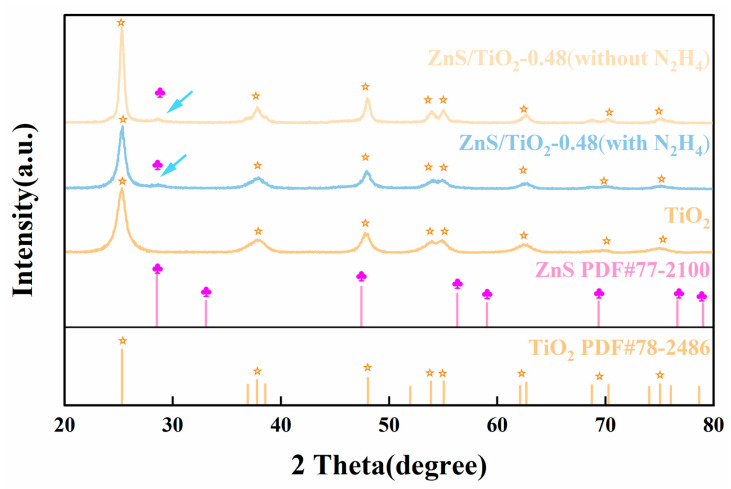
X-ray diffraction (XRD) patterns of pure gel-derived TiO_2_, pure ZnS, ZnS/gel-derived TiO_2_-0.48 (with N_2_H_4_), and ZnS/gel-derived TiO_2_-0.48 (without N_2_H_4_). The orange–yellow pentagrams indicate different crystal planes of TiO_2_, whereas the magenta plum-blossom symbols represent the (111) plane of ZnS.

The phase structure of the ZnS/gel-derived TiO_2_-0.48 composites, with and without hydrazine hydrate (N_2_H_4_) treatment, was examined by X-ray diffraction (XRD), as shown in Figure 1. All samples exhibit diffraction peaks corresponding to anatase gel-derived TiO_2_ (PDF#78-2486) and sphalerite-type ZnS (PDF#77-2100), confirming the formation of the ZnS/TiO_2_ heterostructure without detectable impurity phases or phase transitions. It should be noted that the diffraction features associated with ZnS are intrinsically weak due to its low loading and highly dispersed nature within the gel-derived TiO_2_ matrix. As a result, the ZnS-related reflections are broad and of low intensity in both untreated and N_2_H_4_-treated samples, indicating limited long-range crystalline order. Upon N_2_H_4_ treatment, a slight broadening and attenuation of the diffraction peaks are observed, particularly for the TiO_2_ (101) and ZnS (111) reflections, suggesting increased lattice disorder. Crystallite sizes estimated using the Scherrer equation are therefore regarded as indicative of relative trends rather than absolute values. For the untreated composite, average grain sizes of gel-derived TiO_2_ and ZnS are estimated to be 162 ± 15 nm and 118 ± 7 nm, respectively. In the presence of N_2_H_4_, these values decrease significantly to 102 ± 6 nm (TiO_2_) and 31 ± 1 nm (ZnS). It is important to find that defect formation in ZnS and gel-derived TiO_2_ follows different pathways under N_2_H_4_ treatment. In the case of ZnS, hydrazine influences the nucleation and growth process, leading to suppressed crystallite growth and enhanced lattice disorder. By contrast, gel-derived TiO_2_ undergoes a post-synthetic modification, in which N_2_H_4_ induces surface reduction and oxygen vacancy formation without fundamentally altering the original crystal growth process. This distinction highlights the different roles of N_2_H_4_ in modulating the structural and electronic properties of the two semiconductors. These results suggest that hydrazine treatment modulates the structural coherence of the ZnS/TiO_2_ system, providing a defect-prone framework that is favorable for interfacial charge regulation, rather than inducing a simple phase transformation or crystallinity collapse.

**Figure 2:**
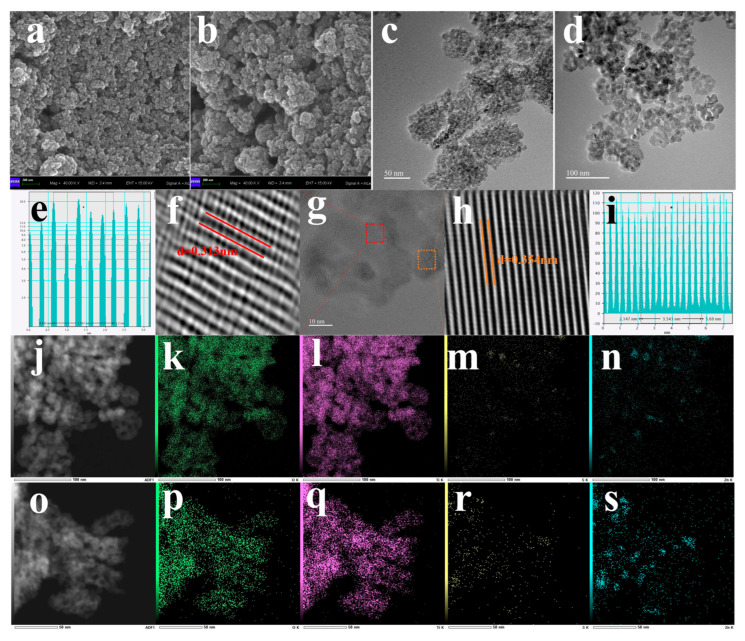
Morphological and structural characterization of the ZnS/gel-derived TiO_2_-0.48 with and without N_2_H_4_ treatment composite. (**a**,**b**) SEM images of ZnS/gel-derived TiO_2_-0.48 with and without N_2_H_4_ treatment composite; (**c**,**d**) TEM images of ZnS/gel-derived TiO_2_-0.48 with and without N_2_H_4_ treatment composite (**e**,**i**) line profile analyses of lattice fringes of ZnS/gel-derived TiO_2_-0.48 with N_2_H_4_ treatment composite; (**f**,**h**) HRTEM images with lattice spacings corresponding to ZnS (111) and TiO_2_ (101) planes of ZnS/gel-derived TiO_2_-0.48 with N_2_H_4_ treatment composite; (**g**) HRTEM images of ZnS/gel-derived TiO_2_-0.48 with N_2_H_4_ treatment composite heterojunction interface zone; (**j**,**o**) HAADF-STEM images of the ZnS/gel-derived TiO_2_ composite. (**k**–**n**) and (**p**–**s**) EDS elemental mapping of Ti, Zn, S, and O of ZnS/gel-derived TiO_2_-0.48 with and without N_2_H_4_ treatment composite, respectively.

The morphology, microstructure, and elemental distribution of ZnS/gel-derived TiO_2_-0.48 composites—prepared with and without hydrazine hydrate (N_2_H_4_)—were systematically characterized using scanning electron microscopy (SEM), transmission electron microscopy (TEM), high-resolution TEM (HRTEM), and energy-dispersive X-ray spectroscopy (EDS), as shown in Figure 2.

The SEM images (Figure 2a,b) reveal that both composites consist of agglomerated nanoparticles with irregular surfaces. The sample treated with N_2_H_4_ displays smaller primary crystallites and a more loosely packed morphology, featuring rougher surfaces and broader interparticle gaps. In contrast, the untreated composite exhibits larger, more compact domains with smoother textures, consistent with the larger crystallite sizes observed by XRD. These morphological differences indicate that hydrazine hydrate effectively regulates crystal growth, leading to nanostructural refinement that favors light scattering and reactant accessibility.

TEM observations (Figure 2c,d) further highlight differences in aggregate architecture. The N_2_H_4_-treated sample appears more transparent and fragmented, suggesting thinner nanosheet-like features with enhanced dispersion. Meanwhile, the untreated composite shows darker contrast and denser particle overlap, pointing to thicker domains and stronger stacking effects. These microstructural differences likely influence interfacial area and charge carrier transport across the heterojunction.

High-resolution lattice imaging (Figure 2f,h) of the N_2_H_4_-treated ZnS/gel-derived TiO_2_-0.48 sample reveals distinct fringe spacings of 0.313 nm and 0.354 nm, which correspond to the (111) plane of ZnS and the (101) plane of TiO_2_, respectively. The clear fringe continuity and sharp phase boundaries support the preservation of crystalline identity and formation of a coherent interface. A representative HRTEM image of the interface region (Figure 2g) shows tight lattice contact without apparent amorphous gaps, confirming successful heterojunction construction. The corresponding FFT-derived line profiles (Figure 2e,i) further validate the observed lattice periodicity.

Elemental distribution maps obtained via EDS (Figure 2j–s) confirm the homogeneous dispersion of Ti, Zn, S, and O throughout both composite systems. The corresponding elemental compositions obtained from EDS analysis are summarized in Table S1. Notably, the sulfur signal in the N_2_H_4_-treated sample is visibly less intense, suggesting a reduction in sulfur content compared to the untreated counterpart. Quantitative EDS analysis shows that the S/Zn atomic ratio decreases from 0.30(untreated) to 0.22(N_2_H_4_-treated), indicating the introduction of sulfur vacancies. Similarly, the O/Ti ratio drops from 2.01 to 1.54, consistent with the formation of oxygen-deficient gel-derived TiO_2_ domains.

These observations collectively demonstrate that hydrazine-assisted synthesis not only reduces grain size and nanosheet thickness, but also promotes the formation of anion vacancies and tight interfacial coupling. Such combined structural and compositional modifications are expected to facilitate charge transfer, inhibit recombination, and enhance CO_2_ activation, thereby contributing to the superior photocatalytic performance of the treated ZnS/gel-derived TiO_2_-0.48 composite.

**Figure 3:**
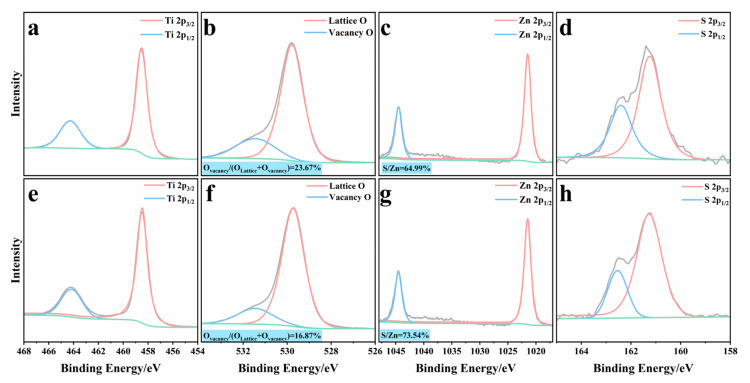
High-resolution XPS spectra of ZnS/gel-derived TiO_2_-0.48 composites with (**a**–**d**) and without (**e**–**h**) N_2_H_4_ treatment: (**a**,**e**) Ti 2p, (**b**,**f**) O 1s, (**c**,**g**) Zn 2p, and (**d**,**h**) S 2p.

The chemical states and surface compositions of ZnS/gel-derived TiO_2_-0.48 composites—with and without hydrazine hydrate (N_2_H_4_) treatment were probed by high-resolution X-ray photoelectron spectroscopy (XPS), as presented in Figure 3. Firstly, XPS survey spectra were further collected to investigate the surface elemental composition of the ZnS/gel-derived TiO_2_-0.48 composites as exhibited in Figure S1. The XPS survey spectra reveal the presence of Ti, O, Zn, and S elements in the ZnS/gel-derived TiO_2_-0.48 composites, and the corresponding surface elemental compositions derived from the survey analysis are summarized in Table S2. After N_2_H_4_ treatment, the S/Zn atomic ratio decreases from 0.70 to 0.61, while the O/Ti ratio decreases from 2.11 to 2.02. Although the absolute atomic ratios differ from those obtained by EDS due to the surface-sensitive nature of XPS, the observed variations indicate a relative depletion of sulfur species in ZnS and a reduced oxygen coordination environment in the near-surface region of gel-derived TiO_2_. Core-level spectra for Ti 2p, O 1s, Zn 2p, and S 2p were recorded to evaluate the oxidation states and defect-related species in both systems. In the Ti 2p region (Figure 3a,e), two well-resolved peaks appear at 458.5 eV (Ti 2p_3/2_) and 464.2 eV (Ti 2p_1/2_), characteristic of Ti^4+^ in anatase-phase gel-derived TiO_2_. No evidence of lower oxidation states (e.g., Ti^3+^) is observed, indicating that the titanium lattice remains chemically stable during the synthetic process. However, a subtle negative shift (~0.2 eV) is noted in the treated sample, suggesting an increased local electron density around Ti centers, likely caused by nearby anion vacancies (e.g., O or S vacancies).

The O 1s spectra (Figure 3b,f) were deconvoluted into two components: lattice oxygen (O_L) at ~529.6 eV and non-lattice oxygen species (O_V), including surface hydroxyl groups and oxygen vacancies, centered near 531.2 eV. The N_2_H_4_-treated composite shows an increased O_V/O_L intensity ratio, which indicates a higher concentration of surface oxygen vacancies or defect-related hydroxyl groups. This trend is consistent with the reduction in the O/Ti atomic ratio from EDS analysis (1.54 vs. 2.01), supporting the occurrence of surface nonstoichiometry.

For the Zn 2p spectra (Figure 3c,g), both samples display typical Zn^2+^ doublets at ~1021.7 eV (Zn 2p_3/2_) and ~1044.8 eV (Zn 2p_1/2_, in line with divalent zinc in ZnS. While the peak positions remain essentially unchanged, the overall Zn signal in the treated sample is slightly attenuated, which may reflect partial surface relaxation or increased structural disorder induced by hydrazine treatment.

The most pronounced difference appears in the S 2p region (Figure 3d,h). Both samples exhibit a pair of peaks at ~161.6 eV and ~162.8 eV, corresponding to the S 2p_3/2_ and S 2p_1/2_ components of sulfide anions (S^2−^). In the N_2_H_4_-treated sample, the S 2p intensity is significantly reduced, with no signals attributable to oxidized sulfur species, such as SO_4_^2−^ or S^0^. This intensity suppression, coupled with the lower S/Zn atomic ratio (0.22 vs. 0.30), provides compelling evidence for the formation of sulfur vacancies (V_S), which are known to modulate electronic structure and facilitate surface charge trapping.

Taken together, the XPS results demonstrate that hydrazine hydrate treatment induces both sulfur and oxygen vacancy formation, without altering the fundamental valence states of the TiO_2_ and ZnS components. These anionic defects are expected to enhance interfacial charge separation and provide additional active sites, thereby contributing to improved photocatalytic efficiency and CH_4_ selectivity in CO_2_ reduction.

### 2.2. Characterization of Photoelectrochemical Test

**Figure 4:**
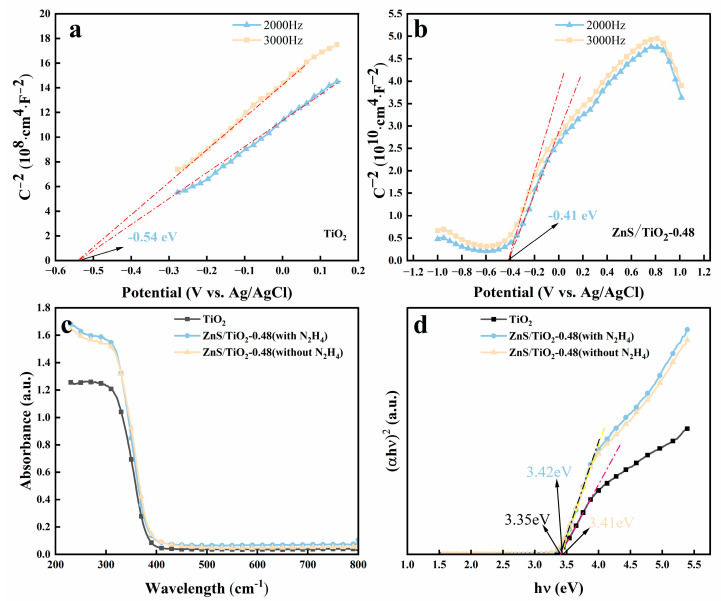
Band structure and optical properties of gel-derived TiO_2_ and ZnS/gel-derived TiO_2_-0.48 composites. (**a**,**b**) Mott–Schottky plots of pure gel-derived TiO_2_ and ZnS/gel-derived TiO_2_-0.48 with N_2_H_4_ treatment; (**c**,**d**) UV–vis absorption spectra and Tauc plots for estimating optical bandgaps of the ZnS/gel-derived TiO_2_-0.48 with and without N_2_H_4_ treatment composite.

The optical response and interfacial electronic properties of the synthesized samples were systematically investigated using Mott–Schottky (MS) analysis and UV–vis diffuse reflectance spectroscopy (DRS), as shown in Figure 4. The MS plots (Figure 4a,b) exhibit linear relationships with positive slopes for both pure gel-derived TiO_2_ and the ZnS/gel-derived TiO_2_-0.48 composite, indicating typical n-type semiconductor behavior when measured at frequencies of 2000 and 3000 Hz. The extrapolated flat-band potentials (V_fb) were determined to be −0.54 V vs. Ag/AgCl for pure gel-derived TiO_2_ and −0.41 V for the N_2_H_4_-treated ZnS/gel-derived TiO_2_-0.48 composite. It should be noted that, in heterojunction systems, the flat-band potential derived from MS analysis does not represent the absolute conduction band edge position of an individual semiconductor component, but rather reflects the effective Fermi level of the composite electrode, which is influenced by interfacial charge redistribution upon heterojunction formation [34]. In this context, a rigorous determination of the band offsets at the ZnS/TiO_2_ interface would require combined ultraviolet photoelectron spectroscopy (UPS) and X-ray photoelectron spectroscopy (XPS) valence band analyses following the Kraut method, which has been widely adopted for quantitative band alignment determination in semiconductor heterojunctions [34,35]. Such measurements are beyond the scope of the present work. Assuming a typical offset of approximately 0.1–0.2 V between the flat-band potential and the conduction band minimum for n-type semiconductors, the apparent conduction band positions can be qualitatively estimated. The observed positive shift of the flat-band potential in the composite relative to pure gel-derived TiO_2_ suggests a redistribution of charge carriers at the ZnS/TiO_2_ interface [36].

The UV–vis absorption spectra (Figure 4c) reveal that all samples possess strong absorption in the ultraviolet region. Upon ZnS loading, a visible-light absorption tail emerges, particularly for the N_2_H_4_-treated ZnS/gel-derived TiO_2_-0.48 composite, which displays an enhanced response in the 350–500 nm range compared to both the untreated composite and pure gel-derived TiO_2_. This enhancement is attributed to defect-state-induced light trapping and possible band structure distortion resulting from sulfur vacancy formation. Tauc plot analysis (Figure 4d), based on the Kubelka–Munk transformation, was employed to estimate the apparent optical band gaps of the samples. The band gap of pure gel-derived TiO_2_ is determined to be 3.35 eV, while the untreated and N_2_H_4_-treated ZnS/gel-derived TiO_2_-0.48 composites exhibit slightly larger apparent band gap values of 3.41 eV and 3.42 eV, respectively. These values should be regarded as apparent band gaps reflecting the dominant absorption edge of the composite system, rather than intrinsic band gaps of the heterojunction interface. Due to the relatively low loading and high dispersion of ZnS, its intrinsic wide band gap (~3.7 eV) contributes weakly to the overall absorption and overlaps with that of TiO_2_, resulting in a single apparent absorption edge in the UV–vis diffuse reflectance spectra. The enhanced visible-light absorption tail observed for the composites is therefore attributed to defect-related sub-bandgap states and light scattering effects induced by interface formation and lattice disorder, rather than a direct narrowing of the intrinsic band gaps.

**Figure 5:**
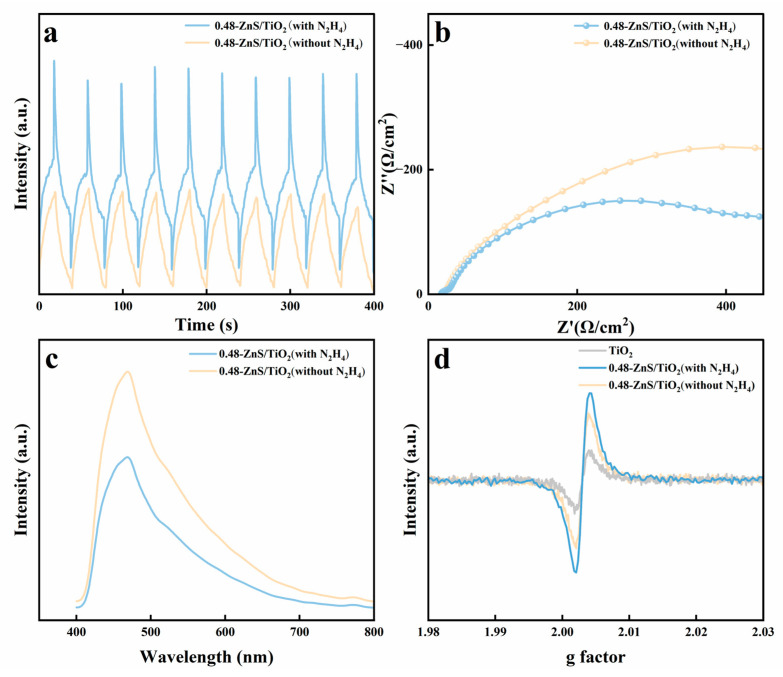
Photoelectrochemical and defect characterization of ZnS/gel-derived TiO_2_-0.48 composites with and without N_2_H_4_ treatment. (**a**) Transient photocurrent responses under chopped light irradiation; (**b**) Nyquist plots from electrochemical impedance spectroscopy; (**c**) photoluminescence spectra; (**d**) EPR spectra showing sulfur/oxygen vacancy signals.

To elucidate the charge carrier dynamics and defect structures in ZnS/gel-derived TiO_2_-0.48 composites with and without N_2_H_4_ treatment, a series of complementary measurements including transient photocurrent, electrochemical impedance spectroscopy (EIS), steady-state photoluminescence (PL), and electron paramagnetic resonance (EPR) were performed, as summarized in Figure 5.

Figure 5a displays the transient photocurrent responses under chopped light irradiation. The N_2_H_4_-treated ZnS/gel-derived TiO_2_-0.48 composite generates a markedly higher and more stable photocurrent than the untreated counterpart. This enhancement in photocurrent intensity reflects improved separation and transport of photoinduced charge carriers, which is attributed to the formation of sulfur vacancies and optimized heterojunction architecture. The rapid rise and decay of current upon light on/off cycles further confirm fast photoresponse and excellent reversibility.

The Nyquist plots (Figure 5b) reveal a significantly smaller semicircle radius for the 0.48-ZnS/TiO_2_ sample with N_2_H_4_ treatment compared with the untreated sample. The charge transfer resistance (Rct), estimated from the diameter of the semicircle, decreases from approximately 245 Ω·cm^2^ for 0.48-ZnS/TiO_2_ (without N_2_H_4_) to about 164 Ω·cm^2^ after N_2_H_4_ treatment, indicating more efficient interfacial charge transfer induced by sulfur vacancy formation. This result aligns with the improved photocurrent response and suggests enhanced electronic conductivity and better interface contact across the heterojunction.

The PL spectra (Figure 5c) were normalized with respect to the corresponding optical absorption to ensure a meaningful comparison of emission intensities among different samples and provide insight into the recombination behavior of photogenerated charge carriers. The untreated composite exhibits a pronounced emission centered at ~470 nm, which is mainly associated with near-band-edge and shallow trap-related radiative recombination in gel-derived TiO_2_. In contrast, the N_2_H_4_-treated ZnS/gel-derived TiO_2_-0.48 composite shows a significantly quenched emission intensity in this spectral region. It should be noted that the PL quenching discussed here primarily refers to the suppression of band-edge-related radiative recombination rather than defect-related visible emission. This behavior indicates enhanced interfacial charge transfer and reduced excitonic recombination due to the introduction of interfacial coupling between ZnS and TiO_2_. Although hydrazine treatment increases the density of defect states, such defects mainly act as charge trapping and transfer sites that facilitate carrier separation, rather than directly enhancing radiative recombination in the observed spectral range.

EPR analysis (Figure 5d) was used to directly identify unpaired electron species associated with paramagnetic defect centers. A prominent signal at g ≈ 2.002 was observed for the N_2_H_4_-treated composite, consistent with the presence of sulfur vacancies (V_S) and/or oxygen vacancies (V_O). The untreated sample exhibits a much weaker signal, while pristine gel-derived TiO_2_ shows negligible paramagnetic response. These findings confirm the effective generation of surface defects during hydrazine-assisted synthesis.

Collectively, these electrochemical and spectroscopic characterizations demonstrate that hydrazine treatment plays a dual role: (i) promoting interfacial charge separation through nanostructural refinement and improved contact, and (ii) introducing abundant sulfur and oxygen vacancies that act as active centers for enhanced carrier trapping and selective multi-electron CO_2_ conversion. These features contribute synergistically to the superior photocatalytic performance observed in ZnS/gel-derived TiO_2_ heterojunction systems.

### 2.3. Photocatalytic CO_2_ Adsorption and Reduction Performance

**Figure 6:**
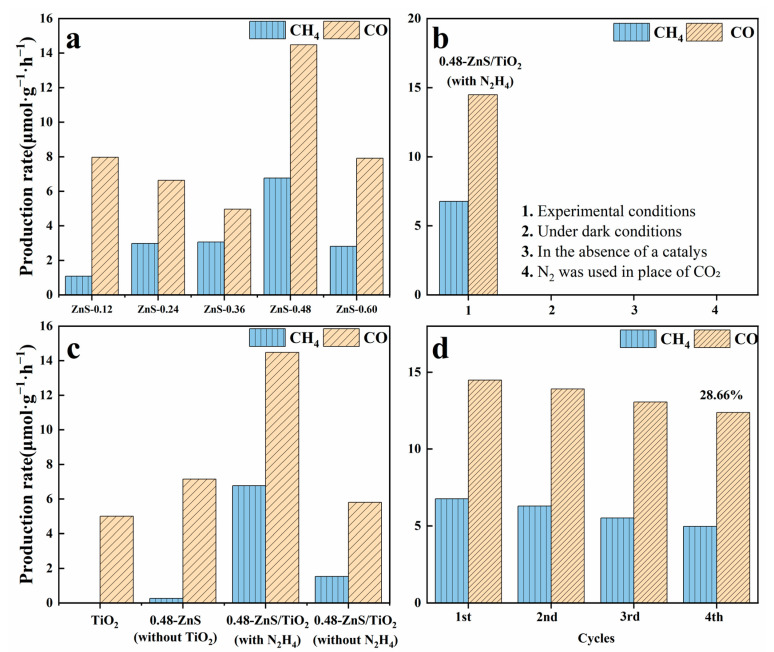
Photocatalytic CO_2_ reduction performance. (**a**) CH_4_ and CO production rates of ZnS/gel-derived TiO_2_ composites with varying ZnS loading. (**b**) Control experiments for photocatalytic CO_2_ reduction performed under dark conditions, in the absence of catalyst, and under an inert atmosphere. (**c**) Comparison among gel-derived TiO_2_, ZnS-0.48 with N_2_H_4_, and ZnS/gel-derived TiO_2_-0.48 with and without N_2_H_4_. (**d**) Cycling stability of ZnS/gel-derived TiO_2_-0.48 composite.

The photocatalytic CO_2_ reduction performance of ZnS/gel-derived TiO_2_ composites—with varying ZnS loadings and hydrazine treatment—was evaluated under simulated solar irradiation. The evolved gaseous products were analyzed using online gas chromatography, as illustrated in Figure 6. Each data point represents the average value of at least three independent measurements. Methane (CH_4_) and carbon monoxide (CO) were identified as the principal products, confirming the occurrence of a multi-electron reduction process over the catalyst surface.

As shown in Figure 6a, the CH_4_ and CO production rates exhibit a clear dependence on ZnS loading. When the ZnCl_2_ precursor amount was gradually increased from 0.12 to 0.48 mmol, the CH_4_ yield steadily improved, peaking at 6.76 μmol·g^−1^·h^−1^ for the ZnS/gel-derived TiO_2_-0.48 sample. However, further increasing the ZnS content to 0.60 mmol resulted in a sharp decline in CH_4_ production, likely due to excessive ZnS coverage blocking gel-derived TiO_2_ active sites or generating charge recombination centers. CO production followed a similar trend, with a maximum rate of 14.47 μmol·g^−1^·h^−1^ observed near the same composition, indicating that optimal heterojunction formation and charge transfer balance are critical for achieving high activity. Notably, although CH_4_ production increases with ZnS loading, the CO yield does not exhibit a proportional increase. This behavior suggests that CO acts as a reaction intermediate and is increasingly consumed via subsequent hydrogenation steps toward CH_4_ on ZnS-rich interfacial/defect sites, rather than desorbing as the final CO product. Therefore, increasing ZnS content primarily shifts the product distribution toward the deep-reduction methanation pathway instead of simply enhancing CO formation. Control experiments were performed to validate the photocatalytic origin of the products (Figure 6b). In the absence of light, catalyst, or CO_2_, no CH_4_ or CO was detected, while significant product generation occurred only under full reaction conditions (light + CO_2_ + catalyst). These results unambiguously confirm that the observed products originate from photocatalytic CO_2_ reduction rather than residual contaminants or thermal side reactions.

To further elucidate the respective contributions of each component and the effect of hydrazine modification, comparative photocatalytic tests were carried out using pure gel-derived TiO_2_, ZnS-only (0.48 mmol), and ZnS/gel-derived TiO_2_-0.48 composites with and without N_2_H_4_ treatment (Figure 6c). Pristine gel-derived TiO_2_ exhibits low overall activity and primarily favors CO formation, reflecting its limited ability to drive multielectron reduction processes. The ZnS-only sample also shows marginal CO_2_ photoreduction activity under the same conditions. In contrast, the ZnS/gel-derived TiO_2_-0.48 composite displays significantly enhanced activity, which is further improved after N_2_H_4_ treatment. Notably, the N_2_H_4_-treated ZnS/gel-derived TiO_2_-0.48 sample achieves the highest CH_4_ yield and overall product formation, outperforming both the untreated composite and the single-component systems. These results indicate that the enhanced CH_4_-selective CO_2_ photoreduction arises from the synergistic interaction at the defect-engineered ZnS/TiO_2_ heterojunction interface. In this system, hydrazine treatment primarily acts as a defect-engineering and interfacial modulation strategy, which promotes charge separation, facilitates intermediate stabilization, and thereby favors the deep-reduction pathway toward CH_4_, rather than solely enhancing the activity of the ZnS component itself.

To further elucidate the individual contributions of each component and the role of hydrazine treatment, comparative photocatalytic tests were performed using pure gel-derived TiO_2_, ZnS-only (0.48 mmol), and ZnS/gel-derived TiO_2_-0.48 composites with and without N_2_H_4_ modification (Figure 6c). Pristine gel-derived TiO_2_ exhibits low overall activity and predominantly produces CO, which can be attributed to its limited capability to sustain multi-electron transfer required for deep CO_2_ reduction. The ZnS-only sample (with N_2_H_4_ treatment) also shows marginal CO_2_ photoreduction activity, indicating that defect-engineered ZnS alone is insufficient to drive efficient methanation. In contrast, the N_2_H_4_-treated ZnS/gel-derived TiO_2_-0.48 composite delivers the highest CH_4_ yield and total product formation, significantly outperforming both the untreated composite and the single-component systems. This pronounced enhancement highlights the essential role of defect–interface synergy in the heterostructure. Specifically, hydrazine treatment introduces sulfur-vacancy-related sites on ZnS and modulates the interfacial electronic structure, while the ZnS/TiO_2_ heterojunction facilitates interfacial charge separation and electron accumulation, jointly promoting CO_2_ activation and the subsequent hydrogenation of CO-derived intermediates toward the deep-reduction CH_4_ pathway.

The operational durability of catalyst was examined through four repeated photocatalytic cycles (Figure 6d). The ZnS/gel-derived TiO_2_-0.48 composite maintained consistent product yields across all runs, with CH_4_ and CO production fluctuating slightly within the ranges of 5.9–6.7 μmol·g^−1^·h^−1^ and 12.8–14.1 μmol·g^−1^·h^−1^, respectively. Despite the low absolute product amounts, the consistent product formation over multiple cycles confirms that the detected signals are reproducible and not attributable to random noise or experimental artifacts. These findings demonstrate the catalysts structural robustness and reaction reproducibility under simulated solar illumination.

In summary, the ZnS/gel-derived TiO_2_-0.48 heterojunction—particularly when modified by hydrazine hydrate—exhibits enhanced photocatalytic CO_2_ reduction efficiency, pronounced CH_4_ selectivity, and long-term stability. These features make it a promising candidate for future solar-to-fuel conversion technologies.

**Figure 7:**
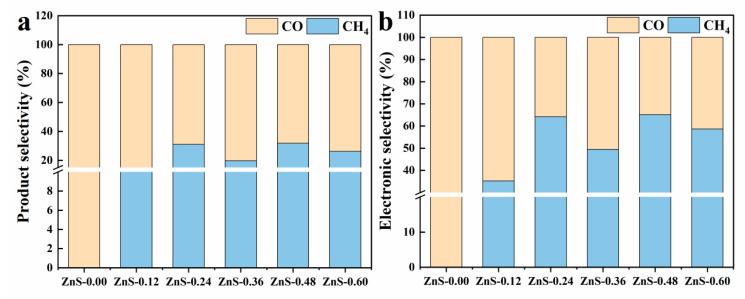
Selectivity analysis of CO_2_ photoreduction over gel-derived TiO_2_ and ZnS/gel-derived TiO_2_ composites. (**a**) Product selectivity for CH_4_ and CO; (**b**) calculated electronic selectivity based on electron consumption for respective products.

To further understand the carbon conversion preference and electron utilization pathways, both product selectivity and electronic selectivity for CO_2_ photoreduction were systematically examined across the prepared catalysts. As shown in Figure 7a, pristine gel-derived TiO_2_ exhibits near-exclusive selectivity toward CO formation, with CH_4_ accounting for less than 10% of the total product distribution. This behavior is attributed to the limited ability of gel-derived TiO_2_ to facilitate the multielectron transfer steps required for CH_4_ evolution. Upon incorporation of ZnS, the CH_4_ proportion increases markedly compared to pristine gel-derived TiO_2_ and reaches a high level at moderate to high ZnS loadings (ZnS/gel-derived TiO_2_-0.24 and 0.48). Among these samples, the ZnS/gel-derived TiO_2_-0.48 composite is identified as the optimal catalyst based on its superior CH_4_ yield and overall photocatalytic performance. At this optimal composition, CH_4_ accounts for 31.7% of the total product yield, indicating that the ZnS domain and its associated surface/interface defects significantly promote deep-level CO_2_ reduction.

Electronic selectivity, calculated based on the number of electrons involved in the formation of each product (8 electrons for CH_4_ and 2 electrons for CO), is presented in Figure 7b as a complementary metric to conventional product selectivity. Consistent with the product distribution trends, gel-derived TiO_2_ exhibits predominant electron consumption toward CO formation (≈84%), whereas the ZnS/gel-derived TiO_2_-0.48 composite shows a higher fraction of electrons directed toward CH_4_ formation (~38.9%). Rather than serving as an independent descriptor, electronic selectivity is used here to further illustrate the preferential allocation of photogenerated electrons toward multi-electron reduction pathways in the defect-engineered heterojunction. This analysis supports the conclusion drawn from product selectivity and yield measurements, namely that the ZnS/gel-derived TiO_2_-0.48 composite achieves an effective balance between CH_4_ yield and electron utilization efficiency, consistent with its superior photocatalytic performance. Therefore, electronic selectivity is employed to reinforce, rather than replace, the conventional selectivity analysis by highlighting the electron-intensive nature of CH_4_ formation.

### 2.4. DFT Calculations and Heterojunction Analysis

To clarify the origin of the enhanced CH_4_ selectivity in ZnS/gel-derived TiO_2_ composites, we combined DFT calculations and band structure characterization to reveal how interfacial energetics and charge dynamics modulate CO_2_ reduction pathways.

**Figure 8:**
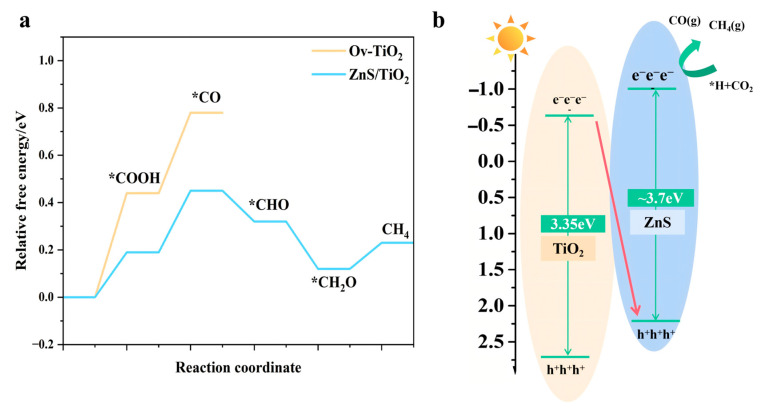
Proposed interfacial charge migration model for the N_2_H_4_-treated ZnS/gel-derived TiO_2_ heterojunction. (**a**) DFT-calculated reaction free energy profiles for CO_2_ photoreduction over Ov–gel-derived TiO_2_ and ZnS/gel-derived TiO_2_ interfaces. (**b**) Band structure diagram of gel-derived TiO_2_ and ZnS/gel-derived TiO_2_ based on experimental flat-band potentials and Tauc-derived bandgaps.

As shown in Figure 8a, the Gibbs free energy profile for CO_2_ photoreduction to CH_4_ was computed on two model surfaces: oxygen-vacancy gel-derived TiO_2_(Ov–TiO_2_) and the ZnS/TiO_2_ heterointerface. The formation energy of the initial protonated intermediate *COOH is significantly lower on ZnS/TiO_2_ (ΔG = +0.21 eV) compared to Ov–TiO_2_ (+0.51 eV), indicating more favorable CO_2_ activation at the heterojunction. Similarly, *CO adsorption is more thermodynamically stabilized on ZnS/TiO_2_ (ΔG = +0.51 eV) than on Ov–TiO_2_ (ΔG = +0.84 eV), and the overall energy span for full CH_4_ formation is narrower. These results suggest that the ZnS/gel-derived TiO_2_ interface facilitates lower-barrier, multielectron transfer steps, thereby enhancing CH_4_ selectivity over the CO-dominated pathway observed on bare gel-derived TiO_2_.

The corresponding band structure analysis is illustrated in Figure 8b based on the flat-band potentials derived from Mott–Schottky measurements and the optical bandgaps obtained from UV–vis diffuse reflectance spectroscopy. Pristine gel-derived TiO_2_ exhibits a flat-band potential of −0.54 V vs. Ag/AgCl and an optical bandgap of 3.35 eV, confirming its typical n-type semiconductor behavior. For the N_2_H_4_-treated ZnS/gel-derived TiO_2_-0.48 composite, a flat-band potential of −0.41 V vs. Ag/AgCl and an optical bandgap of 3.42 eV were obtained. It should be emphasized that, in a multiphase composite system, the flat-band potential extracted from Mott–Schottky analysis represents an apparent flat-band potential of the composite electrode, rather than the intrinsic flat-band potential of pristine ZnS. The measured value can be influenced by the dominant semiconductor/electrolyte interface, surface states, and interfacial charge redistribution. Therefore, it should not be directly used to assign the absolute band edge positions of ZnS. According to well-established literature [17,18], ZnS possesses a wide bandgap (~3.6–3.8 eV) with a much more negative conduction band edge (typically around −0.8 to −1.0 V vs. NHE) than that of TiO_2_. Considering this intrinsic band structure and the work-function-driven Fermi level equilibration at the ZnS/TiO_2_ interface, electrons tend to transfer from ZnS to TiO_2_ upon contact, leading to interfacial band bending and the formation of an internal electric field [17]. Under light irradiation, the relative energetic alignment favors a Z-scheme -like interfacial charge migration pathway, in which photogenerated electrons with strong reductive power are preferentially retained on the ZnS side, while photogenerated holes with strong oxidative power remain on the gel-derived TiO_2_ side. Meanwhile, the less energetic charge carriers recombine at the heterojunction interface. Such a charge migration scenario effectively preserves the redox potentials required for multi-electron CO_2_ reduction and H_2_O oxidation, rather than following a conventional type-II charge transfer process.

Complementary photoelectrochemical results, including enhanced photocurrent response, reduced charge-transfer resistance, and suppressed photoluminescence intensity, consistently indicate efficient interfacial charge separation and prolonged carrier lifetimes. Collectively, these results support a physically reasonable and experimentally consistent Z-scheme-compatible mechanism, which underlies the enhanced and selective CO_2_-to-CH_4_ photoconversion observed for the ZnS/gel-derived TiO_2_ composite.

## 3. Conclusions

In this work, a sulfur-vacancy-rich ZnS/gel-derived TiO_2_ heterojunction photocatalyst was rationally constructed via a hydrazine hydrate-assisted synthesis strategy. Structural characterizations (XRD, HRTEM, and XPS) confirm the formation of a well-defined heterointerface with intimate nanoscale contact and abundant surface sulfur vacancies. Among the investigated samples, the ZnS/gel-derived TiO_2_-0.48 composite exhibits the most pronounced enhancement in photocatalytic performance among the investigated samples, delivering CH_4_ and CO production rates of 7.0 and 14.47 μmol·g^−1^·h^−1^, respectively, with product and electronic selectivities strongly favoring the multi-electron CH_4_ pathway. Mechanistic investigations reveal that hydrazine-induced sulfur vacancies not only introduce localized electronic states that prolong carrier lifetime and suppress charge recombination, but also modulate the interfacial electronic structure to enhance CO_2_ adsorption and activation. Density functional theory calculations demonstrate that the ZnS/gel-derived TiO_2_ interface significantly lowers the energy barriers for *COOH and *CO formation compared to oxygen-vacancy-modified gel-derived TiO_2_, thereby promoting selective CH_4_ evolution. Based on experimental observations and theoretical analysis, a Z-scheme-compatible interfacial charge migration model is proposed, in which strong reductive electrons are preserved on ZnS while strong oxidative holes remain on gel-derived TiO_2_. This work highlights a rational strategy to synergize defect-induced activation and interfacial electronic modulation toward efficient and selective CO_2_ photoreduction, providing useful guidance for the rational design and further optimization of heterostructured photocatalysts for carbon valorization.

## 4. Materials and Methods

### 4.1. Materials

The chemicals used in this study were of analytical grade and were used directly without further purification. Titanium isopropoxide (TTIP), glacial acetic acid, nitric acid (HNO_3_), and isopropyl alcohol (IPA) were purchased from Sinopharm Chemical Reagent Co., Ltd. (Shanghai, China). A sodium hydroxide solution was prepared in the laboratory immediately before use. Thiourea (C_2_H_5_NS, AR, 99%) and zinc chloride (ZnCl_2_, 98%) were obtained from Shanghai Maike Chemical Technology Co., Ltd. (Shanghai, China). Hydrazine hydrate (N_2_H_4_·H_2_O, AR) was supplied by the Sinopharm Chemical Reagent Co., Ltd. (Shanghai, China). Deionized water was used in all experimental steps, including synthesis, dispersion, and washing. Ethanol (C_2_H_5_OH, AR) was purchased from Guangdong Linmin Chemical Reagent Co., Ltd. (Guangzhou, China).

### 4.2. Synthesis of Sol-Gel TiO_2_ and ZnS/Gel-Derived TiO_2_ Composites

Sol–gel TiO_2_ was synthesized with the assistance of microwave heating to improve product homogeneity [37]. In a typical procedure, titanium isopropoxide (4.8 mL) was mixed with glacial acetic acid (0.8 mL) and isopropanol (4 mL) to form a clear precursor solution. Separately, an acidic aqueous phase was prepared by heating a mixture of Milli-Q water (11 mL) and concentrated nitric acid (0.20 mL) to 90 °C. The precursor solution was then poured rapidly into the heated acidic water under strong agitation, producing a uniform milky dispersion. The resulting suspension was transferred into a 60 mL microwave vessel and diluted with deionized water to a total volume of 44 mL. Microwave treatment was carried out at 90 °C for 10 min with continuous stirring at 1200 rpm using a UWave-2000 reactor (Sineo, Shanghai, China). After irradiation, a transparent TiO_2_ hydrosol was obtained. The hydrosol was freeze-dried to yield loosely aggregated TiO_2_ powders.

ZnS-modified TiO_2_ composites were synthesized via a deposition–hydrothermal route. First, gel-derived TiO_2_ (0.96 g) was dispersed in 50 mL of deionized water by ultrasonication for 30 min to obtain a stable suspension (denoted as suspension A). In parallel, aqueous ZnCl_2_ solutions containing different zinc amounts (0.12–0.60 mmol) were prepared in 40 mL of deionized water (solution B). The ZnCl_2_ content was adjusted to obtain a series of samples labeled as TiO_2_/ZnS-x (x = 0.12, 0.24, 0.36, 0.48, and 0.60). Solution B was added to suspension A under continuous stirring, and the mixture was stirred for 20 h to ensure sufficient adsorption of Zn species. Subsequently, 40 mL of NaOH solution (16.8 mM) was introduced dropwise, followed by an additional 20 min of stirring. The resulting precipitate was collected by centrifugation and washed several times with deionized water to remove soluble residues. The washed solid was then redispersed in 60 mL of thioacetamide solution (8 mM) and transferred into a 100 mL Teflon-lined autoclave. Hydrazine hydrate (1 mL, 85 wt%) was added before sealing. The hydrothermal reaction was conducted at 160 °C for 7 h. After cooling to room temperature, the products were separated by centrifugation, rinsed alternately with anhydrous ethanol and deionized water, and finally dried under vacuum at 70 °C. The obtained samples were denoted as ZnS/TiO_2_. Two reference samples were prepared under identical conditions but without the addition of hydrazine hydrate or gel-derived TiO_2_.

### 4.3. Sample Characterization

X-ray diffraction measurements were first carried out to identify the phase composition and crystallinity of the as-prepared samples. The diffraction patterns were recorded on a SmartLab diffractometer (Rigaku, Tokyo, Japan) using Cu Kα radiation (λ = 1.5406 Å), operated at 40 kV and 30 mA. Data were collected over a 2θ range from 5° to 90°, with a step interval of 0.02° and a scanning rate of 10° min^−1^. The microstructural features of the catalysts were subsequently examined by electron microscopy. Scanning electron microscopy (SEM) observations were performed using a Regulus 8100 instrument (Hitachi, Tokyo, Japan). High-resolution transmission electron microscopy (HRTEM) images were acquired on a Talos F200X microscope (Thermo Fisher Scientific, Waltham, MA, USA) operated at an accelerating voltage of 200 kV. For TEM analysis, the samples were ultrasonically dispersed in anhydrous ethanol, deposited onto carbon-coated copper grids, and allowed to dry naturally prior to observation. Photoluminescence spectroscopy was employed to evaluate the recombination behavior of photogenerated charge carriers. The PL spectra were collected on an FLS980 fluorescence spectrometer (Edinburgh Instruments, Livingston, UK) using an excitation wavelength of 325 nm. Photoelectrochemical measurements were conducted using a CHI660D electrochemical workstation (CH Instruments, Shanghai, China) with a standard three-electrode configuration. To fabricate the working electrode, 5 mg of catalyst powder was dispersed in 2 mL of ethanol containing 10 μL of 5 wt% Nafion solution under ultrasonication. An aliquot (100 μL) of the resulting suspension was drop-cast onto an indium tin oxide (ITO) glass substrate with an effective area of 1 cm^2^ and dried at room temperature. A platinum foil and an Ag/AgCl electrode were used as the counter and reference electrodes, respectively. All measurements were performed in 0.5 M Na_2_SO_4_ electrolyte under dark conditions unless otherwise noted.

### 4.4. Evaluation of CO_2_ Photocatalytic Reduction

Photocatalytic CO_2_ reduction experiments were carried out in a closed quartz reactor connected to a CEL-PAEM-D8 photocatalysis analysis system (Au-light, Beijing China Education Technology Co., Ltd., Beijing, China). Prior to the reaction, 7 mg of the photocatalyst was dispersed in 25 mL of deionized water by ultrasonication. The resulting suspension was vacuum-filtered using a Teflon membrane with a pore size below 100 μm, followed by drying at 60 °C under reduced pressure for 30 min to obtain a self-supported catalyst film. The dried membrane was mounted on a stainless-steel holder and placed inside the reactor. To introduce water vapor, 5 mL of deionized water was added to the bottom of the quartz chamber without direct contact with the catalyst. The reactor was then sealed and flushed with high-purity CO_2_ (99.999%) three times to remove residual air, after which the internal pressure was adjusted to approximately 40 kPa. Before illumination, the system was allowed to equilibrate for 30 min using a plunger pump to promote uniform distribution of CO_2_ and water vapor. Illumination was provided by a 300 W xenon lamp (PLS-SXE300+, Au-light, Beijing, China) positioned outside the reactor. During photocatalytic testing, the reaction temperature was maintained at 5 °C by a circulating water-cooling system to suppress thermal effects. Gaseous products generated during the reaction were analyzed online using a gas chromatograph equipped with one thermal conductivity detector and two flame ionization detectors, allowing real-time detection of H_2_, CO, CH_4_, C_2_H_4_, and C_2_H_6_. The detection limits of the gas chromatograph for CH_4_ and CO are on the order of tens of nanomoles, which are well below the amounts detected in the photocatalytic experiments. Moreover, gas chromatography is a well-established and widely accepted technique for quantifying gaseous products in photocatalytic CO_2_ reduction studies, even at trace concentration levels, as demonstrated in numerous previous reports.

### 4.5. Calculation of Product Yield and Selectivity

The formation rates of gaseous products, including H_2_, CO, CH_4_, C_2_H_4_, and C_2_H_6_, were calculated from the amounts determined by gas chromatography. The measured product quantities were normalized with respect to the catalyst loading and the reaction duration. The rate of product formation (R) was calculated using the following equation:

(1) $$R\;=\;\frac{n}{m\;\times \;t}$$

where n represents the amount of product formed (μmol), m is the mass of the photocatalyst (g), and t denotes the illumination time (h). Accordingly, R corresponds to the product yield expressed in μmol g^−1^ h^−1^.

To compare the relative distribution of carbon-based products, the selectivity toward a specific species ($S_{x}$) was defined as the fraction of its molar amount relative to the total amount of carbon-containing reduction products, as given by:

(2) $$S_{x}\;=\;\frac{n_{x}}{\sum n_{i}}\;\times \;100\%$$

where $n_{x}$ is the molar quantity of an individual product and $n_{i}$ is the sum of all carbon-containing products (CO, CH_4_, C_2_H_4_, and C_2_H_6_).

In addition to molar selectivity, electron-based selectivity was introduced to account for the different numbers of electrons involved in forming each product. Taking CH_4_ as an example, the electron selectivity can be expressed as:

(3) $$e^{-}-{\mathrm{Selectivity}}_{{\mathrm{CH}}_{4}}=\frac{8\;\times \;n_{{\mathrm{CH}}_{4}}}{\sum \left(e_{i}\;\times \;n_{i}\right)}\;\times \;100\%$$

where $e_{i}$ denotes the number of electrons required for producing product i (e.g., 2 for CO, 8 for CH_4_, and 12 for C_2_H_4_), and $n_{i}$ is the corresponding molar amount.

All quantitative results were derived from the integrated peak areas of the gas chromatograms using calibration curves established with standard gas mixtures, ensuring accurate determination of product concentrations.

### 4.6. DFT Calculation Method

All first-principles calculations were carried out within the framework of density functional theory using the Vienna Ab initio Simulation Package (VASP, v5.4.4). The core–valence electron interaction was described by the projector augmented-wave (PAW) approach [38,39,40]. A kinetic energy cutoff of 400 eV was adopted for the plane-wave basis set. Electronic convergence was achieved when the total energy change between successive self-consistent steps was smaller than 1 × 10^−5^ eV. Structural optimizations were continued until the residual forces acting on each atom were reduced below 0.03 eV·Å^−1^. To examine the adsorption characteristics of CO_2_ reduction intermediates, surface models were constructed for the TiO_2_(101) facet as well as the ZnS/TiO_2_ interface. Each model consisted of a four-layer slab, in which the lower two layers were fixed to mimic the bulk structure. A vacuum thickness of 15 Å was introduced normal to the surface to avoid interactions between periodically repeated slabs. Reaction energetics were analyzed using Gibbs free energy calculations that account for electronic energies together with vibrational, enthalpic, and entropic contributions. The evaluation of all thermodynamic quantities was performed using the VASPKIT (version 1.3.6) post-processing package [41].

## Data Availability

The original contributions presented in this study are included in the article/Supplementary Material. Further inquiries can be directed to the corresponding authors.

## Supplementary Materials

The following supporting information can be downloaded at: https://www.mdpi.com/article/10.3390/gels12010039/s1, Figure S1: XPS survey spectra of ZnS/gel-derived TiO_2_-0.48 composites (left) without and (right) with N_2_H_4_ treatment. The survey spectra confirm the presence of Ti, O, Zn, and S elements in the composites. The C 1s signal originates from adventitious carbon and was used as a reference for charge correction. Table S1. Elemental compositions of ZnS/gel-derived TiO_2_-0.48 composites with and without N_2_H_4_ treatment determined by EDS analysis. Table S2. Elemental compositions of ZnS/gel-derived TiO_2_-0.48 composites with and without N_2_H_4_ treatment determined by XPS survey analysis.
