# Supplementary material for: Hydrazine-Induced Sulfur Vacancies Promote Interfacial Charge Redistribution in ZnS/Gel-Derived TiO2 for Enhanced CO2 Activation and Methanation
Source: Gels. 2025 Dec 31;12(1):39. doi: 10.3390/gels12010039 (PMC12840932; doi:10.3390/gels12010039)
Supplement: Supplementary file 1 [file gels-12-00039-s001.zip › gels-4042557-supplementary.pdf]

Supplementary material

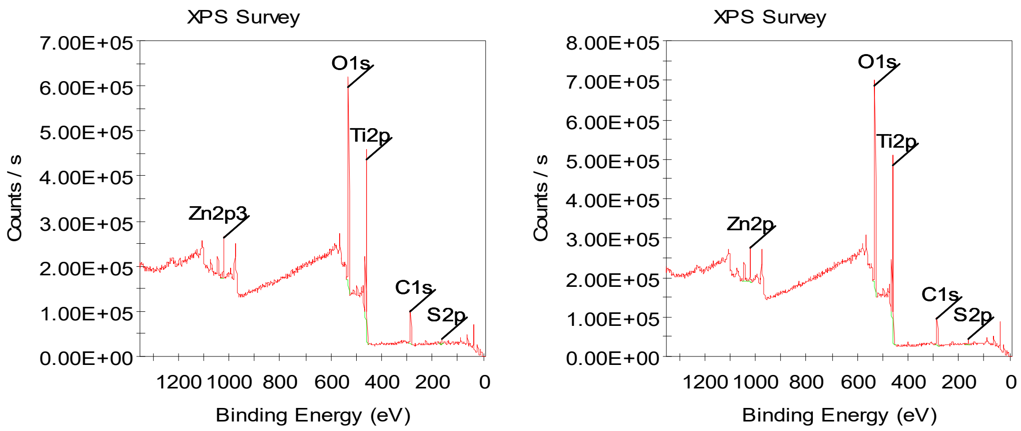

Figure S1. XPS survey spectra of ZnS/gel-derived TiO_2-0.48 composites (left) without and (right) with N_2H_4 treatment. The survey spectra confirm the presence of Ti, O, Zn, and S elements in the composites. The C 1s signal originates from adventitious carbon and was used as a reference for charge correction.

Table S1. Elemental compositions of ZnS/gel-derived TiO_2-0.48 composites with and without N_2H_4 treatment determined by EDS analysis.

Sample	O(at.%)	S(at.%)	Ti(at.%)	Zn(at.%)
ZnS/gel-derived TiO2-0.48	65.7	0.38	32.65	1.27
ZnS/gel-derived TiO_2-0.48(N_2H_4)	57.66	0.92	37.3	4.12

Table S2. Elemental compositions of ZnS/gel-derived TiO_2-0.48 composites with and without N_2H_4 treatment determined by XPS survey analysis.

Sample	O(at.%)	S(at.%)	Ti(at.%)	Zn(at.%)
ZnS/gel-derived TiO2-0.48	63.49	2.64	30.08	3.79
ZnS/gel-derived TiO_2-0.48(N_2H_4)	63.21	2.27	31.27	3.75
